# Thyroid Hormones and Electrocardiographic Parameters: Findings from the Third National Health and Nutrition Examination Survey

**DOI:** 10.1371/journal.pone.0059489

**Published:** 2013-04-12

**Authors:** Yiyi Zhang, Wendy S. Post, Alan Cheng, Elena Blasco-Colmenares, Gordon F. Tomaselli, Eliseo Guallar

**Affiliations:** 1 Department of Epidemiology, Johns Hopkins University Bloomberg School of Public Health, Baltimore, Maryland, United States of America; 2 Welch Center for Prevention, Epidemiology, and Clinical Research, Johns Hopkins Medical Institutions, Baltimore, Maryland, United States of America; 3 Division of Cardiology, Department of Medicine, Johns Hopkins University School of Medicine, Baltimore, Maryland, United States of America; 4 Department of Cardiovascular Epidemiology and Population Genetics, National Center for Cardiovascular Research (CNIC), Madrid, Spain; Universidad Peruana de Ciencias Aplicadas (UPC), Peru

## Abstract

**Introduction:**

Altered thyroid status exerts a major effect on the heart. Individuals with hypo- or hyperthyroidism showed various changes in electrocardiograms. However, little is known about how variations in thyroid hormone levels within the normal range affect electrical activities of the heart in the general population.

**Methods and Results:**

We conducted a cross-sectional analysis of 5,990 men and women from the Third National Health and Nutrition Examination Survey. Serum total T4 was measured by immunoassay and TSH was measured by chemiluminescent assay. We categorized T4 and TSH into 7 groups with cut-offs at the 5^th^, 20^th^, 40^th^, 60^th^, 80^th^, and 95^th^ percentiles of the weighted population distribution. Electrocardiographic parameters were measured from the standard 12-lead electrocardiogram. We found a positive linear association between serum total T4 level and heart rate in men, and a U-shape association between T4 and PR interval in men and women. TSH level was positively associated with QRS interval in men, while a U-shape association between TSH and QRS was observed in women. No clear graded association between thyroid hormones and corrected QT or JT was found, except that men in the highest category of T4 levels appeared to have longer corrected QT and JT, and men in the lowest category of T4 appeared to have shorter corrected QT and JT.

**Conclusions:**

Variation in thyroid hormone levels in the general population, even within the normal range, was associated with various ECG changes.

## Introduction

Thyroid hormones can exert electrophysiologic and inotropic effects on the heart and may modulate the risk of developing atherosclerosis [1]–[4]. Patients with overt hypothyroidism show several electrocardiographic (ECG) changes including sinus bradycardia, low amplitude QRS complexes, QT interval prolongation, and alterations in T wave morphology, while patients with hyperthyroidism can develop atrial arrhythmias such as sinus tachycardia, atrial flutter and atrial fibrillation [5]–[8]. Notably, both prolonged and shortened QT intervals have been reported in hyperthyroidism [9]–[13].

While ECG abnormalities in patients with hypo- and hyperthyroidism are well established, relatively little is known about how variations in thyroid hormone levels within the normal range affect the electrical activity of the heart in the general population [11]–[12]. This study aimed to evaluate the association of total thyroxine (T4) and thyrotropin (TSH) levels with ECG parameters (heart rate, PR interval, QRS duration, QT interval, and JT interval) in a representative sample of the general US population.

## Methods

### Study population

We used data from the Third National Health and Nutrition Examination Survey (NHANES III), a cross-sectional study conducted between 1988 and 1994 that used a multistage stratified clustered probability design to select a representative sample of the civilian non-institutionalized US population. Thyroid hormones were measured in participants 12 years of age and older while 12-lead electrocardiograms (ECGs) were performed on participants 40 years of age and older. Therefore, the present study was restricted to participants 40 years of age and older (N = 8,561) who were eligible for both thyroid function tests and ECG measurements. We excluded 272 participants with missing ECG parameters, 728 participants with missing T4 or TSH, 525 participants with QRS ≥120 ms, and 603 participants with self-report goiter or thyroid disease, or on thyroid medications. In addition, 443 participants with overt or subclinical thyroid disease based on TSH levels were also excluded (413 with TSH>4.5 mU/L, 30 with TSH<0.1 mU/L) [14]. The final analysis was based on 5,990 participants (2,995 men and 2,995 women).

### Data collection

NHANES III included a standardized questionnaire administered in the home by a trained interviewer and a detailed physical examination at a mobile examination center. Demographic characteristics, smoking status, alcohol consumption, medical history, and medication use were assessed during the interview. QT-prolonging medications were defined according to the Arizona Center for Education and Research on Therapeutics database [15]. This database, funded by the Agency for Healthcare Research and Quality (AHRQ), has summarized a list of all the known QT-prolonging drugs. Height and weight were measured and body mass index (BMI) was calculated as weight in kilograms divided by height in meters squared. Blood pressure was measured three times during the in-home interview and three additional times during the at the mobile exam center. Laboratory test results included total cholesterol, HDL cholesterol, serum creatinine, and plasma glucose. Diabetes was defined as a fasting plasma glucose ≥126 mg/dL, a nonfasting plasma glucose ≥200 mg/dL, and/or self-report diabetes with concurrent use of oral hypoglycemic agents or insulin. Estimated glomerular filtration rate (eGFR) was calculated using the abbreviated Modification of Diet in Renal Disease (MDRD) Study formula, re-expressed for standardized serum creatinine [16].

### Measurement of thyroid hormones

Serum was frozen (−20°C) and shipped on dry ice to the Endocrine Services Laboratory, University of Southern California, (Los Angeles, CA) for analysis of T4 and TSH. T4 was measured using an immunoassay (Roche Molecular Biochemicals, Indianapolis, IN) with a reference (normal) range of 57.9 to 169.9 nmol/L. TSH was measured using a chemiluminescence immunometric assay (Nichols Institute Diagnostics, San Juan Capistrano, CA) with a reference (normal) range of 0.39 to 4.6 mU/L.

### ECG parameters

Standard 12-lead resting ECG recordings were performed using a Marquette MAC 12 electrocardiograph (Marquette Medical Systems, Inc., Milwaukee, WI) with signals sampled at 250 Hz per channel. A representative P-QRS-T cycle was then derived by selective averaging using the Dalhousie ECG Analysis Program [17]. The Dalhousie measurement matrix contained several ECG measures of different parts of the ECG waveform, including resting heart rate, PR, QRS, and QT intervals. The JT interval was calculated as QT interval minus QRS interval.

The PR, QT, and JT intervals were adjusted for heart rate, race, and age using the residual method. These adjusted metrics (PRrra, QTrra, and JTrra) were used for subsequent analyses. More specifically, we first regressed the PR, QT, or JT interval, respectively, as dependent variables, on RR interval, race, and age. Adjustment for RR-interval duration was performed using restricted quadratic splines with knots at the 5th, 50th, and 95th percentiles of the overall study population to allow a non-linear relationship of the RR with the PR, QT, or JT intervals. We then obtained model residuals for each participant and centered the residuals at the mean value of the PR, QT, or JT intervals of the study population to facilitate the interpretation of the findings. Residuals are uncorrelated with the regressors (i.e., RR interval, race, and age) and thus adjusted for them. The residuals represent the component of the PR, QT and JT intervals that are not explained by the regressors.

### Statistical analysis

The sampling weights for the ECG component of the survey were used to account for the complex design of NHANES III [18]. All analyses were conducted in men and women separately. To assess the dose-response relationship between thyroid hormone levels and ECG parameters, we categorized the distributions of T4 and TSH into 7 categories with cut-offs at the 5^th^, 20^th^, 40^th^, 60^th^, 80^th^, and 95^th^ percentiles of the weighted population distribution.

We used multivariable linear regression models to estimate adjusted differences and 95% confidence intervals (CIs) for ECG parameters associated with each category of T4 and TSH compared with the middle category. The basic models were adjusted for age, race/ethnicity (non-Hispanic white, non-Hispanic black, Mexican-American, and other), and RR-interval splines (except for the models for heart rate). The full models were further adjusted for BMI, smoking (current, former, and never), alcohol consumption (<12, ≥12 drinks in the past year), systolic blood pressure, blood pressure lowing medication, total and HDL cholesterol, diabetes, history of myocardial infarction, history of congestive heart failure, use of QT-prolonging medications, and creatinine-based eGFR. Finally, we further included T4 and TSH simultaneously in the fully adjusted models. Tests for linear trend were computed by including a variable with the median values of T4 or TSH in each category in the linear regression models. Tests for quadratic trend were computed by using quadratic contrast coefficients.

For sensitivity analyses, we repeated all analyses after excluding participants with conditions that may affect ECG parameters (myocardial infarction, heart failure, use of QT-prolonging medications) or diabetes (final sample size 4,424). All analyses were conducted using SUDAAN (version 10.0.1; Research Triangle Institute, Research Triangle Park, NC).

## Results

The average age of study participants was 54.8 years in men, and 55.8 years in women (Table 1). Average T4 and TSH levels were 107.0 nmol/L and 1.7 mU/L, respectively, in men, and 112.9 nmol/L and 1.9 mU/L, respectively, in women. The cut-offs for T4 and TSH categories were shown in Table 2.

**Table 1:** Baseline characteristics of study participants.

Characteristic	Men (n = 2,995)	Women (n = 2,995)
Age (years)	54.8 (11.6)	55.8 (12.4)
Race		
Non-Hispanic white	81.2	78.4
Non-Hispanic black	8.3	10.1
Mexican-American	3.8	3.5
Systolic blood pressure (mmHg)	128.8 (16.4)	127.2 (19.2)
Total cholestrol (mg/dL)	212.3 (39.0)	220.8 (43.5)
HDL (mg/dL)	45.0 (14.0)	56.1 (16.0)
BMI (kg/m^2^)	27.3 (4.5)	27.1 (6.2)
Estimated-GFR (mL/min/1.73 m^2^)	86.1 (18.9)	84.6 (25.7)
Diabetes	9.1	7.5
Myocardial infarction	6.1	2.6
Congestive heart failure	2.6	1.8
Smoking		
Current	27.9	20.8
Former	44.8	24.8
Never	27.3	54.4
Consume alcohol	59.1	38.3
Use QT prolonging medication	9.6	12.3
Thyroxin (T4, nmol/L)	107.0 (24.7)	112.9 (26.8)
Thyrotropin (TSH, mU/L)	1.7 (0.9)	1.9 (0.9)
ECG parameters		
Heart rate (beat/min)	66.7 (11.3)	69.2 (11.0)
PRrra (ms) *	165.1 (26.4)	159.3 (25.3)
QRS (ms)	99.6 (9.3)	93.5 (9.2)
QTrra (ms) *	403.1 (18.4)	407.5 (18.7)
JTrra (ms) *	303.5 (18.1)	314.0 (18.3)

Values are means (SD) or percentages unless otherwise noted.

* . PRrra, QTrra, JTrra: RR-interval, race-, and age corrected PR, QT, and JT intervals.

**Table 2:** Percentiles and cut-offs of T4 and TSH distribution.

Percentiles (%)	Cut-offs	Men (n = 2,995)	Women (n = 2,995)
**T4 (nmol/L)**			
1. [0, 5)	[0,70.2)	142	114
2. [5, 20)	[70.2, 89.6)	490	359
3. [20, 40)	[89.6, 103.2)	680	543
4. [40, 60)	[103.2, 114.7)	605	628
5. [60, 80)	[114.7, 129.1)	589	631
6. [80, 95)	[129.1, 150.9)	389	509
7. ≥95	≥ 150.9	100	211
**TSH (mU/L)**			
1. [0, 5)	[0.1, 0.6)	195	156
2. [5, 20)	[0.6, 1.0)	445	384
3. [20, 40)	[1.0, 1.4)	611	583
4. [40, 60)	[1.4, 1.8)	640	622
5. [60, 80)	[1.8, 2.4)	558	568
6. [80, 95)	[2.4, 3.6)	435	518
7. ≥95	[3.6, 4.5]	111	164

**Figure 1:**
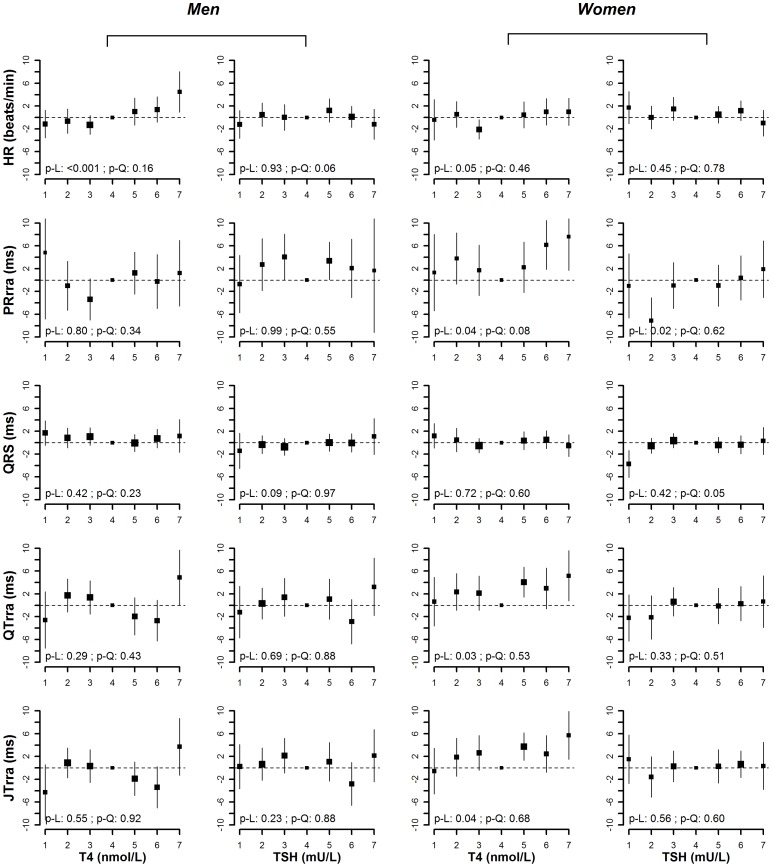
Age, race/ethnicity, RR-interval adjusted means (95% CI) of heart rate, PRrra interval, QRS duration, QTrra and JTrra interval by categories of T4 and TSH (p-L denotes p-value for linear trend, and p-Q denotes p-value for quadratic trend).

**Figure 2:**
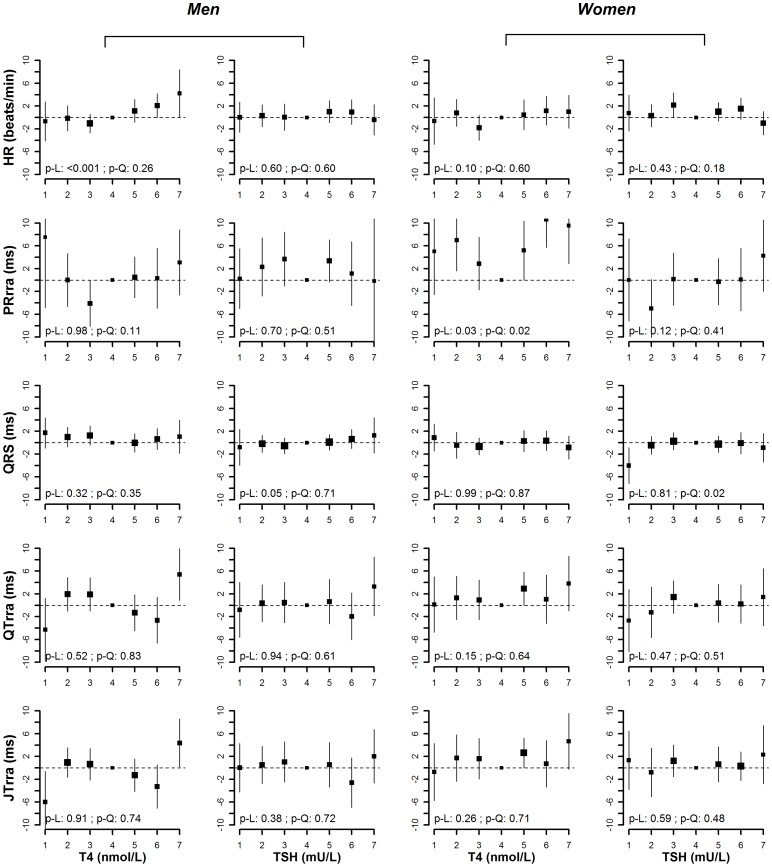
Multivariate adjusted means (95% CI) of heart rate, PRrra interval, QRS duration, QTrra and JTrra interval by categories of T4 and TSH (p-L denotes p-value for linear trend, and p-Q denotes p-value for quadratic trend). Models were adjusted for age, race/ethnicity (non-Hispanic white, non-Hispanic black, Mexican-American, and other), RR-interval splines (except for the models of heart rate), BMI, smoking (current, former, and never), alcohol consumption (<12, ≥12 drinks in the past year), systolic blood pressure, blood pressure lowing medication, total and HDL cholesterol, diabetes, history of myocardial infarction, history of congestive heart failure, use of QT-prolonging medications, and creatinine-based eGFR.

After adjusting for age, race/ethnicity and RR-interval, T4 levels were positively associated with heart rate in men and women (p-value for linear trend <0.001 in men and 0.05 in women) (Figure 1). In the fully adjusted model, the positive association between T4 and heart rate was still evident in men (p-value for linear trend <0.001), but not in women (p-value for linear trend  = 0.10) (Figure 2). The average difference in heart rate comparing the highest vs. the lowest category of T4 was 4.9 beat/min (95% CI 0.6 to 9.2) in men and 1.7 beat/min (−2.7 to 6.0) in women.

We found a U-shaped association between T4 levels and PRrra interval in women (p-value for quadratic trend 0.08 in the basic model and 0.02 in the fully adjusted model). In the fully adjusted model, the average difference in PRrra interval comparing the lowest vs. the middle category of T4 was 5.0 ms (−2.5 to 12.6), and the average difference comparing the highest vs. the middle category was 9.6 ms (2.9 to 16.2). There was a similar U-shaped pattern between T4 levels and PRrra in men, but the p-values for quadratic trend did not reach statistical significance (p-value for quadratic trend 0.34 in the basic model and 0.11 in the fully adjusted model).

There was a marginally positive linear association between TSH levels and QRS interval in men (p-value for linear trend 0.09 in the basic model and 0.05 in the fully adjusted model), but a U-shaped association in women (p-value for quadratic trend 0.05 in the basic model and 0.02 in the fully adjusted model). In the fully adjusted model, the average difference in QRS duration comparing the highest vs. the lowest category of TSH in men was 2.1 ms (−2.1 to 6.2). In women, the average difference in QRS duration comparing the lowest vs. the middle category of TSH was −4.0 ms (−7.2 to −0.9) and the average difference comparing the highest vs. the middle category was −0.9 ms (−3.4 to 1.6).

In models adjusted for age, race/ethnicity and RR-interval, T4 levels were positively associated with QTrra and JTrra intervals in women (p-value for linear trend  = 0.03 for QTrra, and 0.04 for JTrra), but the associations became non-significant in the fully adjusted models (p-value for linear trend 0.15 for QTrra, and 0.26 for JTrra). In men, the association between T4 levels with QTrra or JTrra intervals was not significant, although men in the highest category of T4 appeared to have longer QTrra and JTrra intervals and men in the lowest category of T4 appeared to have shorter QTrra and JTrra intervals compard to the rest of the groups. In men, the average difference in QTrra and JTrra intervals comparing the lowest vs. the middle category of T4 in the fully adjusted models were −4.3 ms (−9.8 to 1.3) and −6.0 ms (−11.4 to −0.6), respectively, while the average difference in QTrra and JTrra intervals comparing the highest vs. the middle category of T4 were 5.4 ms (0.8 to 10.0) and 4.4 ms (0.2 to 8.6).

Finally, including T4 and TSH in the same model (Appendix S1) and exclusion of individuals with diabetes, myocardial infarction, heart failure, or taking QT-prolonging medications (Appendix S2) resulted in similar findings.

## Discussion

This analysis of NHANES III men and women with thyroid hormone levels within the normal range showed a positive linear association between serum T4 levels and heart rate in men and a U-shaped association between T4 levels and PR interval in men and women. In addition, TSH levels were marginally positively associated with QRS interval in men, but showed a U-shaped association with QRS interval in women. There was no consistent association between thyroid hormone levels and QT or JT intervals, except that men in the highest category of T4 levels appeared to have longer QT and JT intervals and men in the lowest category of T4 levels appeared to have shorter QT and JT intervals compared to the middle group.

Tachycardia and bradycardia are well known signs of hyper- and hypothyroidism, respectively [9], [13], [19]–[24]. Experimental data suggested that the faster heart rate in hyperthyroidism was partly due to the regulatory effect of thyroid hormones on sodium pump density and enhancement of Na+ and K+ currents [25]. Thyroid hormones can increase heart rate by increasing sinus node automaticity, decreasing the action potential duration and the refraction period of the atrial myocardium as well as the atrioventricular nodal refraction period [25]. In addition, increased sympathetic and decreased vagal modulation in hyperthyroidism could also increase the heart rate [26].

Less is known, however, about how thyroid hormone levels within the normal range affect heart rate in the general population. The Study of Health in Pomerania (1,748 men and 1,862 women in northeast Germany) seems to be the only previous population-based study that has investigated this question. In this study, there was no significant association between TSH levels and heart rate [12]. We found, however, that T4 was positively related to heart rate in men, but not in women. This gender difference has not been described previously in the literature and warrants confirmation in other population-based studies.

The effect of thyroid hormones on the QT interval duration is controversial. A majority of animal and clinical studies have reported a prolonged heart rate-corrected QT interval (QTc) in hypothyroidism [22], [27]–[32]. The underlying mechanism may be decreasing *I_Ks_*, or an increased sympathetic influence on the autonomic cardiovascular system (i.e., elevated catecholamine levels, particularly norepinephrine release from sympathetic nerves) as shown in some studies [9], [31], [33]. In contrast, while several animal models have reported a shortened repolarization time in the hyperthyroid state [27], [34], small studies in patients with hyperthyroidism have reported prolonged QTc intervals [9]–[10], [13]. A study comparing 16 hyperthyroid patients with Graves' disease with matched healthy controls found significant prolongations in the 24-h average QTc in hyperthyroid patients, with positive correlations of free T3 and free T4 with QTc [9]. Another study reported significant increases in QTc interval in 32 patients with subclinical hyperthyroidism compared to healthy controls, but QTc was not correlated with free T4 or TSH levels [10]. In addition, QTc intervals significantly decreased in 47 patients with hyperthyroidism after achievement of euthyroid state, however this may be mainly driven by the changes in heart rate [13].

Limited data from population-based studies were also inconsistent. The Study of Health in Pomerania showed a positive association between TSH levels and QTc intervals [12]. Another study of 939 elderly men and women in Netherlands without clinical or subclinical hypothyroidism found no significant association between TSH and QTc, but they reported a positive linear association between free T4 and QTc duration in men but not in women [11]. Our analysis of NHANES data suggested no clear graded association between thyroid hormones and corrected QT or JT intervals. However, we found that men in the highest category of T4 levels appeared to have longer corrected QT and JT intervals compared to the middle group, and men in the lowest category of T4 appeared to have shorter corrected QT and JT intervals. These observations were still evident after including T4 and TSH simultaneously in the model. The different findings may partly explained by the differences in population characteristics, choices of QT correction formula, or selection of cut-points for T4 and TSH. Future studies are necessary to validate our findings in other population-based samples.

Finally, very few studies have evaluated the association of thyroid hormone levels with other electrocardiographic parameters such as PR interval or QRS duration. Experimental studies in hypothyroid dogs have reported both no changes in PR or QRS duration [35], and prolonged P wave and QRS duration compared to euthyroid dogs [32]. In humans, a study of 18 patients with hypothyroidism found no significant changes in the PR and QRS duration after L-thyroxine treatment [29], and a study of 25 patients with hyperthyroidism reported no difference in PR interval compared to healthy controls [20]. Our results showed a U-shaped relationship between T4 and PR interval in both men and women. We also found a linear association between TSH levels and QRS durations in men and a U-shaped association in women. Again, additional studies are needed to confirm these observations.

Several limitations of this study need to be considered. Firstly, the cross-sectional design limited our ability to make statements regarding the causality of the relation between thyroid hormone levels and ECG parameters. Secondly, we only had available measurements of total T4, but not of free T4 or T3 measurements in NHANES III. T3 is much more potent than T4, and we might expect stronger associations if we would have used T3 in the analysis. Furthermore, most of the circulating thyroid hormone in the blood is bound to transport proteins and only a very small fraction is free (unbound) and biologically active. Therefore, the measurement of total T4 levels may not represent the actual level of free (active) T4. Finally, thyroid hormones and ECG measurements were performed only at a single time at baseline, which may result in non-differential measurement error as there might be substantial within person variability. Repeated measurements may be needed to reduce measurement error and better characterize the association between thyroid status and ECG changes in future studies.

## Conclusion

In conclusion, data from NHANES III suggested that variation in thyroid hormone levels within the normality range in the general population was associated with various ECG changes, including a linear association between serum total T4 level and heart rate in men, a U-shaped association between T4 and PR interval in men and women, a linear association between TSH and QRS duration in men, and a U-shaped association between TSH and QRS in women. In addition, men in the highest category of T4 had longer QT and JT intervals, although there was no clear graded association between thyroid hormone levels and QT or JT intervals. Further research is needed to confirm these findings and to elucidate the underlying mechanisms, especially those related to gender differences in the effect of thyroid hormones on ECG parameters.

## Supporting Information

Appendix S1 **Multivariate adjusted means (95% CI) of heart rate, PRrra interval, QRS duration, QTrra and JTrra interval by categories of T4 and TSH (p-L denotes p-value for linear trend, and p-Q denotes p-value for quadratic trend).** Models were adjusted for age, race/ethnicity (non-Hispanic white, non-Hispanic black, Mexican-American, and other), RR-interval splines (except for the models of heart rate), BMI, smoking (current, former, and never), alcohol consumption (<12, ≥12 drinks in the past year), systolic blood pressure, blood pressure lowing medication, total and HDL cholesterol, diabetes, history of myocardial infarction, history of congestive heart failure, use of QT-prolonging medications, creatinine-based eGFR, and T4 (in the models for TSH), and TSH (in the models for T4).
(TIF)

Appendix S2 **Multivariate adjusted means (95% CI) of heart rate, PRrra interval, QRS duration, QTrra and JTrra interval by categories of T4 and TSH, excluding participant with diabetes, myocardial infarction, heart failure, or taking QT-prolonging medications (p-L denotes p-value for linear trend, and p-Q denotes p-value for quadratic trend).** Models were adjusted for for age, race/ethnicity (non-Hispanic white, non-Hispanic black, Mexican-American, and other), RR-interval splines (except for the models of heart rate), BMI, smoking (current, former, and never), alcohol consumption (<12, ≥12 drinks in the past year), systolic blood pressure, blood pressure lowing medication, total and HDL cholesterol, diabetes, history of myocardial infarction, history of congestive heart failure, use of QT-prolonging medications, and creatinine-based eGFR.
(TIF)
