# Supplementary figures and images for: Thyroid Hormones and Electrocardiographic Parameters: Findings from the Third National Health and Nutrition Examination Survey
Source: PLoS One. 2013 Apr 12;8(4):e59489. doi: 10.1371/journal.pone.0059489 (PMC3625180; doi:10.1371/journal.pone.0059489)

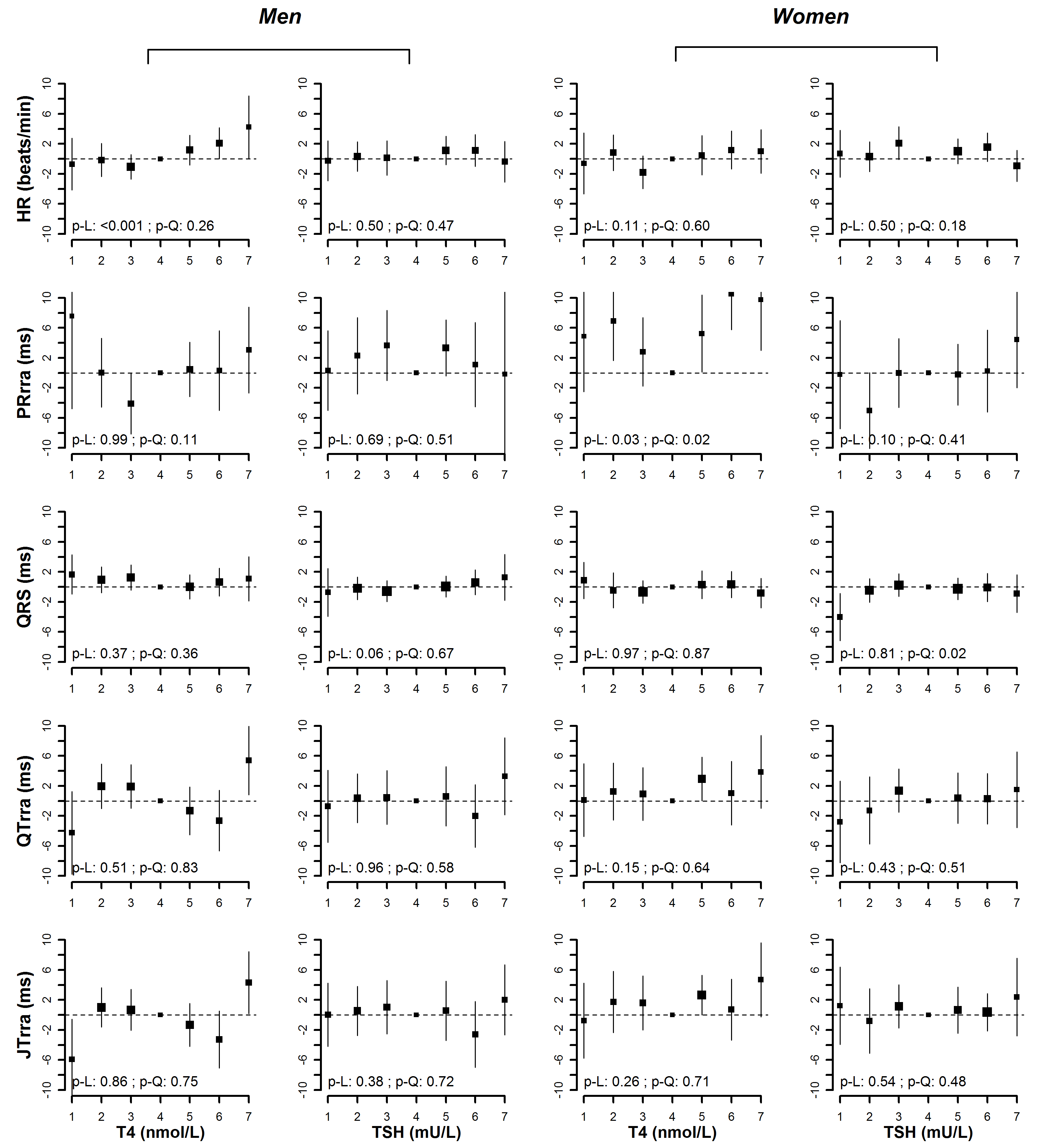

Supplement: Appendix S1 — Multivariate adjusted means (95% CI) of heart rate, PRrra interval, QRS duration, QTrra and JTrra interval by categories of T4 and TSH (p-L denotes p-value for linear trend, and p-Q denotes p-value for quadratic trend). Models were adjusted for age, race/ethnicity (non-Hispanic white, non-Hispanic black, Mexican-American, and other), RR-interval splines (except for the models of heart rate), BMI, smoking (current, former, and never), alcohol consumption (<12, ≥12 drinks in the past year), systolic blood pressure, blood pressure lowing medication, total and HDL cholesterol, diabetes, history of myocardial infarction, history of congestive heart failure, use of QT-prolonging medications, creatinine-based eGFR, and T4 (in the models for TSH), and TSH (in the models for T4). (TIF) [file pone.0059489.s001.tif]

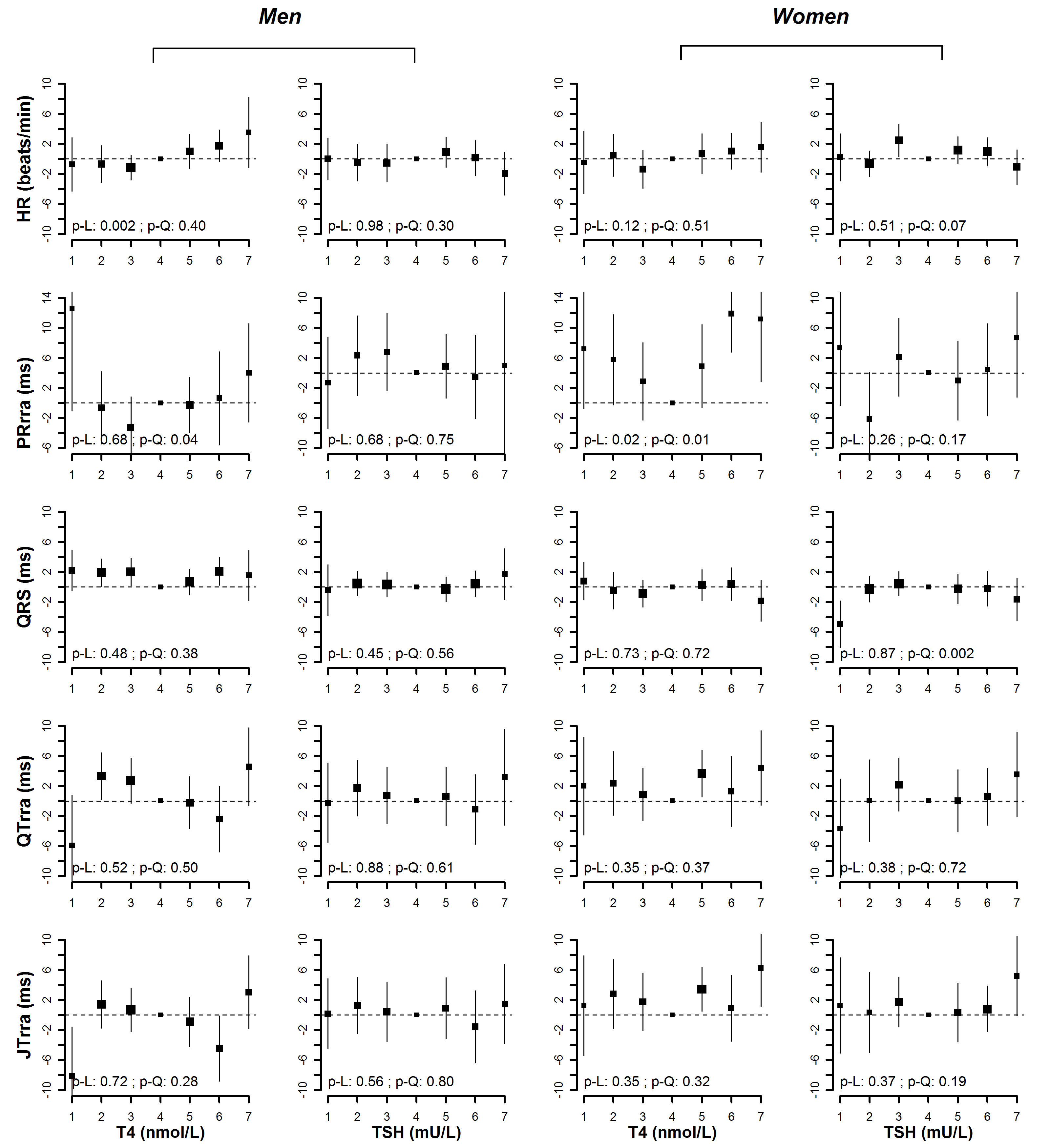

Supplement: Appendix S2 — Multivariate adjusted means (95% CI) of heart rate, PRrra interval, QRS duration, QTrra and JTrra interval by categories of T4 and TSH, excluding participant with diabetes, myocardial infarction, heart failure, or taking QT-prolonging medications (p-L denotes p-value for linear trend, and p-Q denotes p-value for quadratic trend). Models were adjusted for for age, race/ethnicity (non-Hispanic white, non-Hispanic black, Mexican-American, and other), RR-interval splines (except for the models of heart rate), BMI, smoking (current, former, and never), alcohol consumption (<12, ≥12 drinks in the past year), systolic blood pressure, blood pressure lowing medication, total and HDL cholesterol, diabetes, history of myocardial infarction, history of congestive heart failure, use of QT-prolonging medications, and creatinine-based eGFR. (TIF) [file pone.0059489.s002.tif]
